# Supplementary material for: Dynamic Prediction of an Event Using Multiple Longitudinal Markers: A Model Averaging Approach
Source: Stat Med. 2025 Jun 3;44(13-14):e70122. doi: 10.1002/sim.70122 (PMC12134451; doi:10.1002/sim.70122)
Supplement: Supplementary file 1 — Data S1. Supporting Information. [file SIM-44-0-s001.pdf]

Supplementary Materials

Dynamic Prediction of an Event Using Multiple Longitudinal Markers: A Model Averaging Approach

Reza Hashemi

Department of Statistics, Razi University, Kermanshah, Iran.

Taban Baghfalaki

Department of Mathematics, The University of Manchester, Manchester, UK

Inserm, Research Center U1219, Univ. Bordeaux, ISPED, F33076 Bordeaux, France.

Viviane Philipps

Inserm, Research Center U1219, Univ. Bordeaux, ISPED, F33076 Bordeaux, France.

Helene Jacqmin-Gadda

Inserm, Research Center U1219, Univ. Bordeaux, ISPED, F33076 Bordeaux, France.

Supplementary Material A: Results of application section

Table A. 1: The estimated regression coefficients of the time-to-death sub-model from the all-marker joint model on PBC2 data. Est: posterior mean, SD: standard deviation, 2.5% CI: lower bound of credible interval and 97.5% CI: upper bound of credible interval.

	Est	SD	2.5% CI	97.5% CI
Age	0.050	0.011	0.028	0.071
Drug	-0.188	0.213	-0.600	0.234
Spiders	-0.025	0.009	-0.043	-0.007
Albumin	-1.731	0.405	-2.529	-0.920
log(Alkaline)	0.395	0.319	-0.210	1.026
log(SGOT)	-0.303	0.347	-0.952	0.392
log(Platelets)	-0.384	0.279	-0.937	0.161
log(Prothrombin)	4.140	1.542	1.102	7.103
log(SerBilir)	0.934	0.170	0.600	1.280

Table A. 2: The estimated regression coefficients and variance of errors of the longitudinal sub-model from the all-marker joint model on PBC2 data. Est: posterior mean, SD: standard deviation, 2.5% CI: lower bound of credible interval and 97.5% CI: upper bound of credible interval.

	Est	SD	2.5% CI	97.5% CI
Intercept (β_{01})	-1.092	0.124	-1.348	-0.851
Time (β_{11})	0.039	0.108	-0.180	0.238
Intercept (β_{02})	3.546	0.023	3.501	3.589
Time (β_{12})	-0.099	0.007	-0.112	-0.086
Intercept (β_{03})	6.292	0.890	4.681	7.215
Time (β_{13})	0.181	0.232	-0.053	0.645
Intercept (β_{04})	4.704	0.026	4.649	4.754
Time (β_{14})	0.018	0.007	0.005	0.032
Intercept (β_{05})	5.463	0.023	5.418	5.507
Time (β_{15})	-0.067	0.005	-0.078	-0.057
Intercept (β_{06})	2.361	0.005	2.352	2.371
Time (β_{16})	0.018	0.001	0.016	0.021
Intercept (β_{07})	0.501	0.059	0.381	0.616
Time (β_{17})	0.174	0.015	0.144	0.203
σ_1	0.317	0.006	0.306	0.328
σ_2	0.932	0.626	0.318	2.024
σ_3	0.274	0.005	0.264	0.285
σ_4	0.209	0.004	0.201	0.217
σ_5	0.070	0.001	0.068	0.073
σ_6	0.332	0.006	0.320	0.346

Table A. 3: The estimated covariance matrix of the random effects from the all-marker joint model on PBC2 data.

	b_{01}	b_{11}	b_{02}	b_{12}	b_{03}	b_{13}	b_{04}	b_{14}	b_{05}	b_{15}	b_{06}	b_{16}	b_{07}	b_{17}
b_{01}	4.124	0.350	-0.138	-0.021	0.058	0.029	0.137	0.022	-0.158	-0.009	0.054	0.000	0.635	0.027
b_{11}	0.350	2.641	-0.185	-0.041	0.392	0.037	0.201	0.052	-0.075	-0.047	0.049	0.005	0.594	0.145
b_{02}	-0.138	-0.185	0.118	0.005	-0.120	-0.008	-0.041	-0.005	0.038	0.006	-0.012	-0.002	-0.183	-0.019
b_{12}	-0.021	-0.041	0.005	0.003	-0.014	-0.003	-0.008	-0.003	0.004	0.001	-0.002	-0.000	-0.025	-0.007
b_{03}	0.058	0.392	-0.120	-0.014	0.921	-0.038	0.193	0.015	0.029	-0.015	0.012	0.002	0.443	0.048
b_{13}	0.029	0.037	-0.008	-0.003	-0.038	0.018	0.000	0.004	-0.004	-0.001	0.002	0.000	0.019	0.010
b_{04}	0.137	0.201	-0.041	-0.008	0.193	0.000	0.184	0.005	-0.012	-0.009	0.009	0.001	0.279	0.021
b_{14}	0.022	0.052	-0.005	-0.003	0.015	0.004	0.005	0.004	-0.001	-0.001	0.002	0.000	0.024	0.010
b_{05}	-0.158	-0.075	0.038	0.004	0.029	-0.004	-0.012	-0.001	0.144	0.003	-0.011	-0.001	-0.087	-0.008
b_{15}	-0.009	-0.047	0.006	0.001	-0.015	-0.001	-0.009	-0.001	0.003	0.003	-0.002	-0.000	-0.024	-0.004
b_{06}	0.054	0.049	-0.012	-0.002	0.012	0.002	0.009	0.002	-0.011	-0.002	0.005	0.000	0.044	0.006
b_{16}	0.000	0.005	-0.002	-0.000	0.002	0.000	0.001	0.000	-0.001	-0.000	0.000	0.000	0.003	0.001
b_{07}	0.635	0.594	-0.183	-0.025	0.443	0.019	0.279	0.024	-0.087	-0.024	0.044	0.003	1.015	0.066
b_{17}	0.027	0.145	-0.019	-0.007	0.048	0.010	0.021	0.010	-0.008	-0.004	0.006	0.001	0.066	0.029

Table A. 4: AUC and Brier score computed by 5-fold cross-validation for landmark times $s = 0, 2, 4, 6, 8, 10$ and prediction windows of 2 years for the comparison between the one-marker joint models, the two-marker joint models, the all-marker joint model, the one-marker MA and the two-marker MA for PBC2 data.

	s=0	s=2	s=4	s=6	s=8	s=10
	$\widehat{AUC}(s, 2)$					
Spiders	0.801	0.731	0.765	0.689	0.654	0.629
Albumin	0.844	0.784	0.830	0.790	0.878	0.838
log(Alkaline)	0.679	0.693	0.680	0.673	0.562	0.522
log(SGOT)	0.751	0.808	0.750	0.808	0.669	0.736
log(Platelets)	0.762	0.635	0.704	0.603	0.719	0.677
log(Prothrombin)	0.871	0.798	0.714	0.702	0.738	0.789
log(SerBilir)	0.853	0.923	0.861	0.878	0.773	0.869
Spiders, Albumin	0.860	0.811	0.843	0.822	0.862	0.799
Spiders, log(Alkaline)	0.774	0.770	0.761	0.761	0.629	0.605
Spiders, log(SGOT)	0.794	0.834	0.788	0.844	0.671	0.731
Spiders, log(Platelets)	0.825	0.730	0.787	0.675	0.716	0.640
Spiders, log(Prothrombin)	0.876	0.807	0.726	0.717	0.753	0.793
Spiders, log(SerBilir)	0.862	0.925	0.855	0.885	0.756	0.864
Albumin, log(Alkaline)	0.831	0.828	0.838	0.815	0.870	0.758
Albumin, log(SGOT)	0.828	0.866	0.839	0.831	0.814	0.835
Albumin, log(Platelets)	0.852	0.801	0.829	0.781	0.895	0.790
Albumin, log(Prothrombin)	0.888	0.862	0.830	0.781	0.866	0.857
Albumin, log(SerBilir)	0.867	0.933	0.885	0.878	0.833	0.892
log(Alkaline), log(SGOT)	0.739	0.808	0.757	0.813	0.677	0.730
log(Alkaline), log(Platelets)	0.782	0.771	0.775	0.719	0.676	0.597
log(Alkaline), log(Prothrombin)	0.850	0.836	0.751	0.742	0.748	0.743
log(Alkaline), log(SerBilir)	0.849	0.922	0.852	0.888	0.786	0.899
log(SGOT), log(Platelets)	0.790	0.834	0.775	0.805	0.727	0.760
log(SGOT), log(Prothrombin)	0.844	0.869	0.769	0.825	0.724	0.799
log(SGOT), log(SerBilir)	0.852	0.920	0.853	0.882	0.781	0.874
log(Platelets), log(Prothrombin)	0.877	0.796	0.734	0.683	0.777	0.789
log(Platelets),log(SerBilir)	0.867	0.933	0.867	0.875	0.784	0.883
log(Prothrombin), log(SerBilir)	0.876	0.928	0.857	0.878	0.784	0.903
one-marker MA	0.875	0.922	0.850	0.879	0.880	0.826
two-marker MA	0.883	0.935	0.862	0.878	0.881	0.832
All-marker JM	0.883	0.937	0.881	0.874	0.858	0.893
landmarking	-	0.886	0.745	0.665	0.624	0.653
	$\widehat{BS}(s, 2)$					
Spiders	0.094	0.133	0.088	0.099	0.135	0.135
Albumin	0.081	0.121	0.083	0.094	0.087	0.113
log(Alkaline)	0.101	0.128	0.087	0.097	0.128	0.141
log(SGOT)	0.096	0.112	0.089	0.082	0.122	0.123
log(Platelets)	0.095	0.138	0.098	0.098	0.114	0.121
log(Prothrombin)	0.079	0.120	0.092	0.089	0.120	0.090
log(SerBilir)	0.081	0.078	0.073	0.060	0.124	0.110
Spiders, Albumin	0.081	0.117	0.080	0.095	0.093	0.119
Spiders, log(Alkaline)	0.095	0.125	0.084	0.096	0.134	0.139
Spiders, log(SGOT)	0.093	0.108	0.085	0.085	0.126	0.123
Spiders, log(Platelets)	0.093	0.129	0.092	0.097	0.128	0.124
Spiders, log(Prothrombin)	0.079	0.119	0.090	0.090	0.119	0.091
Spiders, log(SerBilir)	0.079	0.079	0.072	0.061	0.127	0.116
Albumin, log(Alkaline)	0.086	0.113	0.082	0.092	0.090	0.115
Albumin, log(SGOT)	0.084	0.104	0.085	0.080	0.096	0.109
Albumin, log(Platelets)	0.081	0.119	0.084	0.094	0.085	0.115
Albumin, log(Prothrombin)	0.067	0.108	0.084	0.088	0.092	0.098
Albumin, log(SerBilir)	0.074	0.073	0.069	0.058	0.103	0.088
log(Alkaline), log(SGOT)	0.097	0.111	0.088	0.082	0.121	0.125
log(Alkaline), log(Platelets)	0.099	0.120	0.091	0.090	0.118	0.133
log(Alkaline), log(Prothrombin)	0.084	0.112	0.086	0.084	0.117	0.097
log(Alkaline), log(SerBilir)	0.080	0.080	0.074	0.061	0.125	0.104
log(SGOT), log(Platelets)	0.094	0.107	0.091	0.079	0.113	0.120
log(SGOT), log(Prothrombin)	0.082	0.103	0.088	0.077	0.115	0.099
log(SGOT), log(SerBilir)	0.082	0.081	0.075	0.060	0.122	0.106
log(Platelets), log(Prothrombin)	0.078	0.119	0.095	0.091	0.115	0.091
log(Platelets),log(SerBilir)	0.077	0.076	0.071	0.060	0.118	0.100
log(Prothrombin), log(SerBilir)	0.072	0.077	0.072	0.059	0.122	0.090
one-marker MA	0.074	0.078	0.078	0.060	0.087	0.117
two-marker MA	0.067	0.075	0.075	0.062	0.087	0.114
All-marker JM	0.068	0.076	0.072	0.058	0.092	0.077
landmarking	-	0.092	0.088	0.118	0.195	0.146

Table A. 5: Dynamic prediction and standard deviation computed using 5-fold cross-validation for landmark times $s = 0, 2, 4, 6, 8, 10$ and prediction windows of 2 years for the comparison of the five best prediction models for randomly selected individuals from PBC2 data.

		$s=0$		$s=2$		$s=4$		$s=6$		$s=8$		$s=10$	
id		Est.	SD	Est.	SD	Est.	SD	Est.	SD	Est.	SD	Est.	SD
6	All-marker JM	0.015	0.015	0.020	0.021	0.030	0.035	0.063	0.059	-	-	-	-
	Albumin, log(SerBilir)	0.020	0.015	0.021	0.015	0.023	0.019	0.032	0.037	-	-	-	-
	Two-marker MA	0.062	0.036	0.031	0.024	0.040	0.055	0.047	0.051	-	-	-	-
	One-marker MA	0.084	0.126	0.038	0.031	0.053	0.039	0.062	0.062	-	-	-	-
	log(Prothrombin), log(SerBilir)	0.054	0.029	0.055	0.026	0.063	0.035	0.082	0.061	-	-	-	-
35	All-marker JM	0.057	0.031	0.130	0.099	0.273	0.235	0.486	0.307	-	-	-	-
	Albumin, log(SerBilir)	0.039	0.026	0.103	0.077	0.255	0.209	0.429	0.313	-	-	-	-
	two-marker MA	0.063	0.035	0.122	0.088	0.255	0.197	0.445	0.294	-	-	-	-
	One-marker MA	0.088	0.046	0.178	0.092	0.274	0.223	0.508	0.279	-	-	-	-
	log(Prothrombin), log(SerBilir)	0.077	0.035	0.182	0.097	0.343	0.197	0.483	0.240	-	-	-	-
44	All-marker JM	0.031	0.013	0.058	0.018	0.104	0.031	0.203	0.061	0.375	0.145	-	-
	Albumin, log(SerBilir)	0.017	0.010	0.029	0.014	0.053	0.026	0.113	0.057	0.216	0.120	-	-
	Two-marker MA	0.039	0.027	0.029	0.015	0.049	0.024	0.108	0.054	0.343	0.196	-	-
	One-marker MA	0.077	0.044	0.063	0.015	0.099	0.026	0.138	0.035	0.337	0.171	-	-
	log(Prothrombin), log(SerBilir)	0.024	0.013	0.033	0.017	0.049	0.023	0.079	0.039	0.123	0.067	-	-
127	All-marker JM	0.007	0.004	0.013	0.005	0.023	0.010	0.045	0.027	0.098	0.071	0.193	0.137
	Albumin, log(SerBilir)	0.004	0.002	0.006	0.003	0.011	0.006	0.021	0.012	0.043	0.028	0.064	0.048
	Two-marker MA	0.011	0.010	0.006	0.004	0.014	0.008	0.028	0.015	0.059	0.031	0.114	0.076
	One-marker MA	0.031	0.016	0.022	0.005	0.039	0.019	0.074	0.016	0.071	0.053	0.161	0.079
	log(Prothrombin), log(SerBilir)	0.004	0.002	0.007	0.003	0.013	0.006	0.025	0.012	0.049	0.027	0.085	0.054
196	All-marker JM	0.082	0.038	0.154	0.060	0.294	0.118	0.549	0.212	-	-	-	-
	Albumin, log(SerBilir)	0.050	0.021	0.084	0.035	0.133	0.061	0.249	0.128	-	-	-	-
	Two-marker MA	0.194	0.133	0.103	0.045	0.171	0.121	0.258	0.129	-	-	-	-
	One-marker MA	0.171	0.144	0.073	0.021	0.184	0.107	0.180	0.061	-	-	-	-
	log(Prothrombin), log(SerBilir)	0.074	0.025	0.128	0.039	0.225	0.079	0.385	0.144	-	-	-	-
206	All-marker JM	0.012	0.006	0.011	0.006	0.013	0.008	0.018	0.018	0.033	0.044	-	-
	Albumin, log(SerBilir)	0.008	0.004	0.008	0.004	0.010	0.005	0.011	0.009	0.018	0.014	-	-
	Two-marker MA	0.026	0.016	0.009	0.004	0.014	0.008	0.016	0.010	0.068	0.051	-	-
	One-marker MA	0.050	0.046	0.026	0.006	0.041	0.023	0.035	0.015	0.094	0.070	-	-
	log(Prothrombin), log(SerBilir)	0.016	0.008	0.020	0.008	0.025	0.012	0.036	0.022	0.051	0.041	-	-
278	All-marker JM	0.116	0.064	0.314	0.162	0.561	0.256	-	-	-	-	-	-
	Albumin, log(SerBilir)	0.103	0.052	0.312	0.190	0.580	0.294	-	-	-	-	-	-
	Two-marker MA	0.078	0.047	0.348	0.191	0.419	0.308	-	-	-	-	-	-
	One-marker MA	0.075	0.045	0.241	0.117	0.344	0.257	-	-	-	-	-	-
	log(Prothrombin), log(SerBilir)	0.074	0.030	0.193	0.097	0.410	0.214	-	-	-	-	-	-

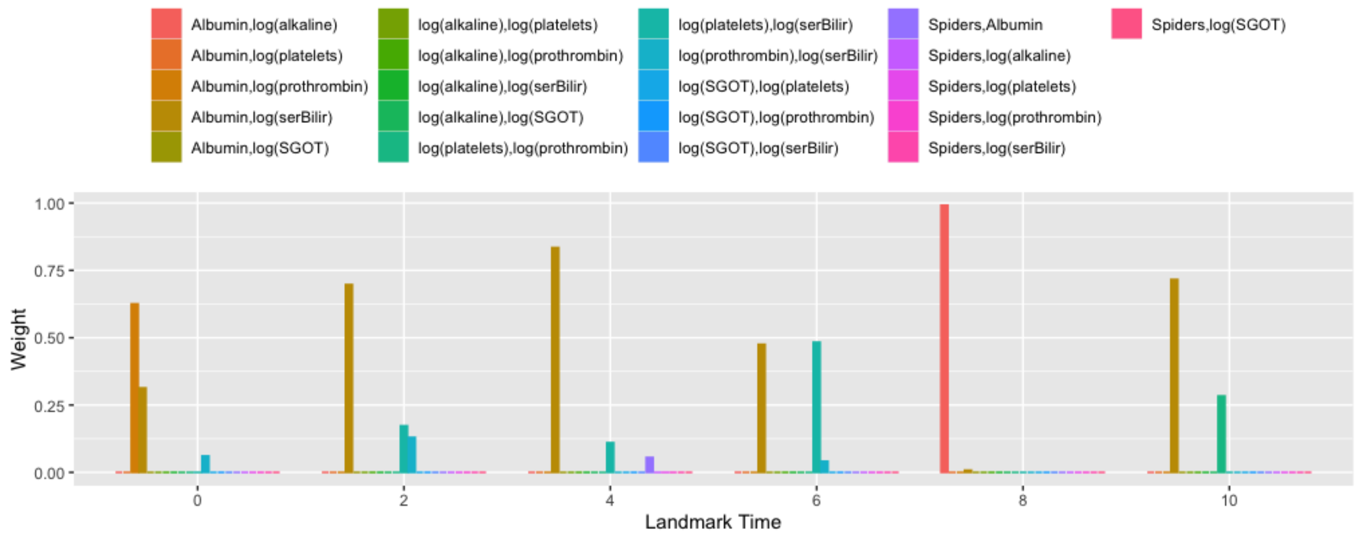

Figure A. 1: The distribution of weights for the landmark times $s = 0, 2, 4, 6, 8, 10$ years and prediction windows of 2 years for the two-marker MA using PBC2 data.

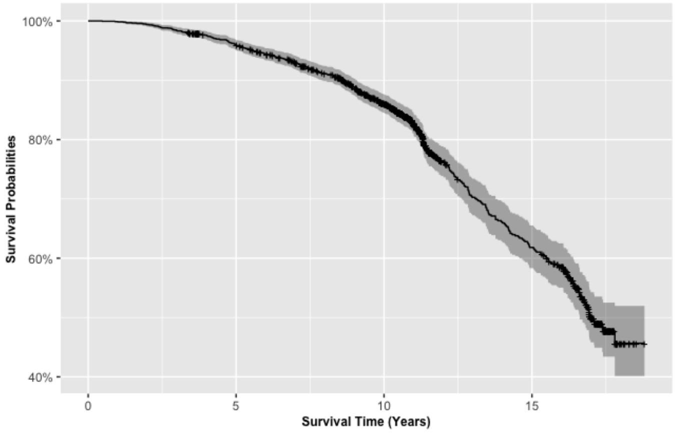

Figure A. 2: The Kaplan-Meier survival function for death over 17 years of follow-up in the 3C cohort.

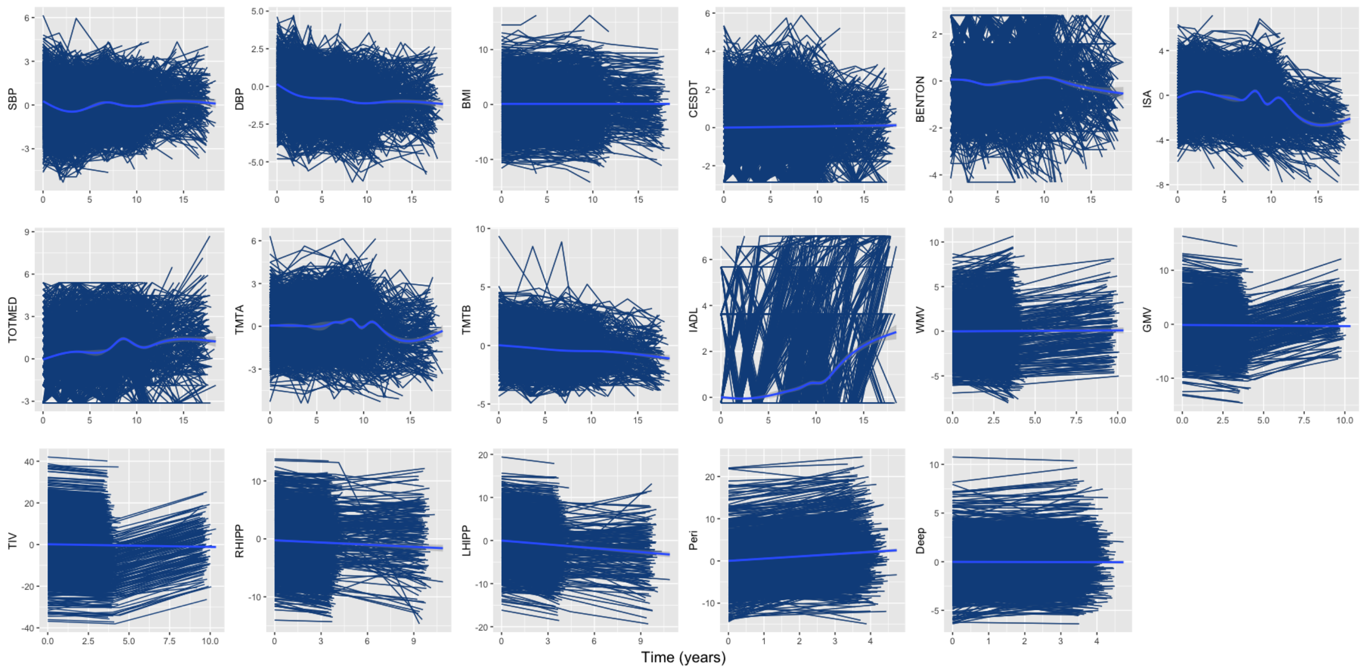

Figure A. 3: Individual trajectories from the normalized clinical, neuropsychological and imaging longitudinal markers in the 3C study. Systolic blood pressure (SBP), diastolic blood pressure (DBP), body mass index (BMI), depressive symptomatology measured using the Center for Epidemiologic Studies Depression scale (CESDT), the visual retention test of Benton (BENTON), Isaac Set Test (ISA), the total number of medications (TOTMED), the trail making test A and B (TMTA and TMTB), functional dependency assessed using Instrumental Activity of Daily Living scale (IADL), white matter volume (WMV), gray matter volume (GMW), total intracranial volume (TIV), right and left hippocampal volume (RHIPP and LHIPP), for the 5-year risk of dementia of thevolumes of White Matter Hyperintensities in the periventricular (Peri) and deep (Deep) white matter on the 3C data.

Table A. 6: The estimated association parameters of the time-to-death sub-model from the one-marker joint models on 3C data. Est: posterior mean, SD: standard deviation, 2.5% CI: lower bound of credible interval, and 97.5% CI: upper bound of credible interval.

	Est	SD	2.5% CI	97.5% CI
WMV	0.001	0.020	-0.038	0.042
GMV	-0.070	0.018	-0.105	-0.036
TIV	-0.007	0.007	-0.020	0.006
HIPPR	-0.061	0.013	-0.087	-0.036
HIPPL	-0.043	0.011	-0.064	-0.019
Peri	0.005	0.007	-0.007	0.018
Deep	0.038	0.028	-0.015	0.093
SBP	0.033	0.064	-0.092	0.158
DBP	-0.021	0.057	-0.133	0.086
BMI	-0.011	0.014	-0.037	0.018
CESDT	0.264	0.054	0.163	0.372
BENTON	-0.418	0.087	-0.593	-0.245
ISA	-0.195	0.038	-0.267	-0.115
TOTMED	0.121	0.043	0.038	0.205
TMTA	-0.215	0.047	-0.307	-0.122
TMTB	-0.246	0.058	-0.361	-0.131
IADL	0.248	0.036	0.180	0.312

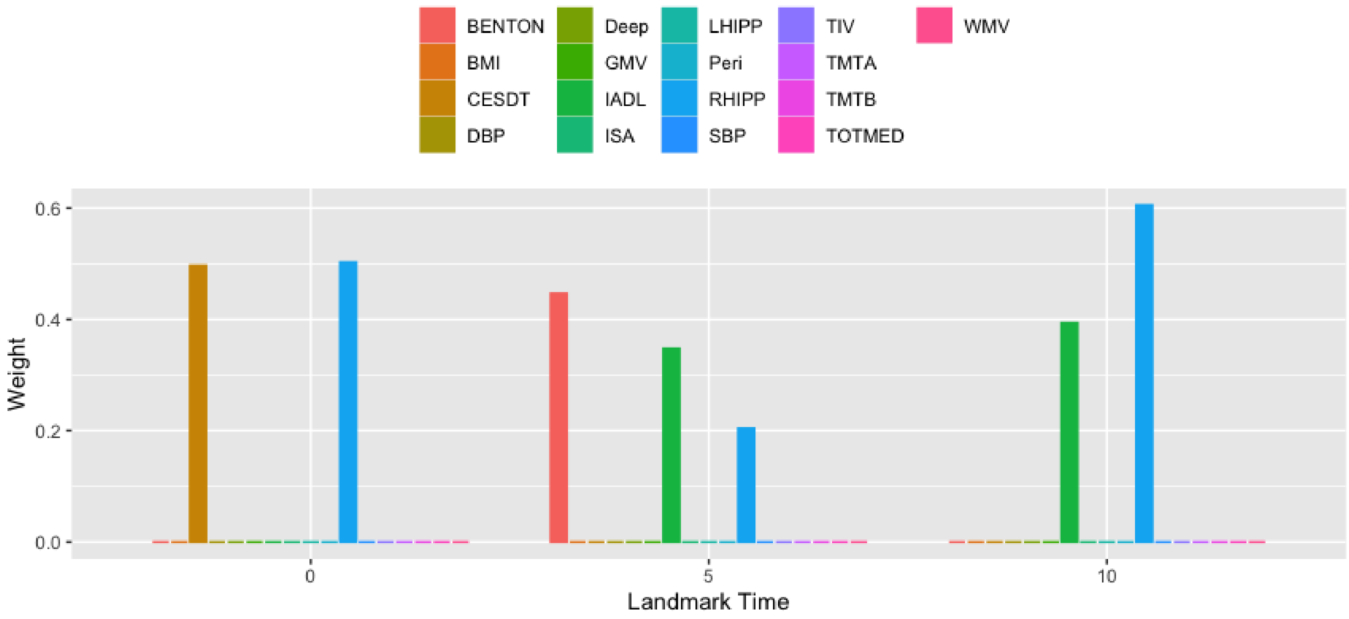

Figure A. 4: The weights distribution for the landmark times $s = 0, 5, 10$ years and prediction windows of 5 years for the MA in 3C data.

Supplementary Material B: Results of simulation studies

Table B. 1: Simulation results for Scenario 1 (I.1) with $\boldsymbol{\alpha} = (-0.5, -0.5, -0.5)$ and independent markers. Mean and standard deviation of $\widehat{AUC}(s,t)$ and $\widehat{MSE}(s,t)$ for $s = 0, 0.5, 1, 1.5$ and $t = 0.5$ over 100 replications.

		$s=0$		$s=0.5$		$s=1$		$s=1.5$	
		Mean	SD	Mean	SD	Mean	SD	Mean	SD
$\widehat{AUC}(s,t)$	Marker 1	0.654	0.055	0.676	0.049	0.649	0.048	0.634	0.053
	Marker 2	0.621	0.069	0.665	0.050	0.661	0.058	0.661	0.062
	Marker 3	0.646	0.061	0.654	0.045	0.672	0.044	0.654	0.060
	Markers 1,2	0.689	0.063	0.744	0.047	0.741	0.044	0.737	0.060
	Markers 1,3	0.708	0.051	0.736	0.041	0.742	0.041	0.729	0.047
	Markers 2,3	0.685	0.056	0.728	0.041	0.750	0.050	0.746	0.045
	One-marker MA	0.689	0.050	0.749	0.053	0.783	0.047	0.776	0.052
	Two-marker MA	0.726	0.055	0.786	0.041	0.815	0.038	0.825	0.038
	All-marker JM	0.736	0.055	0.795	0.036	0.823	0.038	0.843	0.037
	Real value	0.747	0.052	0.803	0.035	0.830	0.041	0.849	0.036
$\widehat{MSE}(s,t)$	Marker 1	0.010	0.002	0.043	0.004	0.087	0.008	0.164	0.020
	Marker 2	0.010	0.002	0.043	0.006	0.087	0.010	0.162	0.019
	Marker 3	0.010	0.002	0.043	0.006	0.086	0.009	0.160	0.016
	Markers 1,2	0.006	0.001	0.027	0.003	0.054	0.008	0.109	0.019
	Markers 1,3	0.006	0.002	0.027	0.003	0.052	0.006	0.106	0.014
	Markers 2,3	0.006	0.001	0.027	0.004	0.052	0.007	0.104	0.011
	One-marker MA	0.009	0.002	0.039	0.005	0.076	0.008	0.150	0.017
	Two-marker MA	0.005	0.001	0.021	0.003	0.038	0.005	0.088	0.012
	All-marker JM	0.001	0.000	0.006	0.001	0.013	0.003	0.037	0.007

Table B. 2: Simulation results for Scenario 1 (I.2) with $\boldsymbol{\alpha} = (0, -0.5, -0.5)$ and independent markers. Mean and standard deviation of $\widehat{AUC}(s,t)$ and $\widehat{MSE}(s,t)$ for $s = 0, 0.5, 1, 1.5$ and $t = 0.5$ over 100 replications.

		$s = 0$		$s = 0.5$		$s = 1$		$s = 1.5$	
		Mean	SD	Mean	SD	Mean	SD	Mean	SD
$\widehat{AUC}(s,t)$	Marker 1	0.511	0.073	0.482	0.058	0.499	0.063	0.496	0.070
	Marker 2	0.613	0.069	0.661	0.064	0.698	0.056	0.676	0.052
	Marker 3	0.627	0.073	0.660	0.057	0.684	0.051	0.681	0.061
	Markers 1,2	0.613	0.069	0.660	0.065	0.697	0.056	0.676	0.052
	Markers 1,3	0.628	0.074	0.658	0.058	0.683	0.050	0.680	0.063
	Markers 2,3	0.663	0.075	0.726	0.055	0.788	0.052	0.796	0.040
	One-marker MA	0.639	0.081	0.717	0.056	0.773	0.054	0.771	0.048
	Two-marker MA	0.658	0.076	0.725	0.054	0.787	0.051	0.795	0.040
	All-marker JM	0.662	0.075	0.726	0.055	0.788	0.052	0.795	0.042
	Real value	0.673	0.077	0.732	0.058	0.789	0.050	0.799	0.041
$\widehat{MSE}(s,t)$	Marker 1	0.005	0.001	0.024	0.005	0.063	0.009	0.136	0.014
	Marker 2	0.004	0.001	0.015	0.003	0.039	0.006	0.091	0.010
	Marker 3	0.003	0.001	0.015	0.003	0.039	0.005	0.092	0.011
	Markers 1,2	0.004	0.001	0.015	0.003	0.039	0.006	0.092	0.010
	Markers 1,3	0.003	0.001	0.015	0.003	0.039	0.005	0.093	0.012
	Markers 2,3	0.001	0.000	0.003	0.001	0.008	0.002	0.030	0.007
	One-marker MA	0.003	0.001	0.013	0.003	0.032	0.005	0.083	0.010
	Two-marker MA	0.001	0.001	0.004	0.001	0.010	0.003	0.031	0.007
	All-marker JM	0.001	0.000	0.003	0.001	0.008	0.002	0.031	0.007

Table B. 3: Simulation results for Scenario 1 (I.3) with $\boldsymbol{\alpha} = (0, -0.5, -1)$ and independent markers. Mean and standard deviation of $\widehat{AUC}(s,t)$ and $\widehat{MSE}(s,t)$ for $s = 0, 0.5, 1, 1.5$ and $t = 0.5$ over 100 replications.

		$s = 0$		$s = 0.5$		$s = 1$		$s = 1.5$	
		Mean	SD	Mean	SD	Mean	SD	Mean	SD
$\widehat{AUC}(s,t)$	Marker 1	0.490	0.059	0.498	0.054	0.498	0.056	0.499	0.066
	Marker 2	0.607	0.052	0.612	0.047	0.603	0.059	0.581	0.060
	Marker 3	0.729	0.054	0.764	0.043	0.767	0.044	0.753	0.057
	Markers 1,2	0.604	0.054	0.613	0.046	0.602	0.059	0.582	0.059
	Markers 1,3	0.728	0.054	0.763	0.043	0.766	0.045	0.753	0.056
	Markers 2,3	0.753	0.053	0.802	0.040	0.827	0.040	0.821	0.052
	One-marker MA	0.733	0.055	0.771	0.042	0.790	0.043	0.771	0.058
	Two-marker MA	0.751	0.054	0.801	0.040	0.825	0.039	0.821	0.052
	All-marker JM	0.753	0.052	0.801	0.041	0.826	0.041	0.822	0.051
	Real value	0.763	0.054	0.807	0.040	0.833	0.039	0.827	0.051
$\widehat{MSE}(s,t)$	Marker 1	0.027	0.005	0.085	0.011	0.147	0.013	0.254	0.021
	Marker 2	0.025	0.005	0.077	0.010	0.129	0.012	0.227	0.019
	Marker 3	0.011	0.003	0.032	0.005	0.054	0.008	0.113	0.014
	Markers 1,2	0.025	0.005	0.077	0.010	0.129	0.012	0.227	0.019
	Markers 1,3	0.011	0.003	0.032	0.005	0.054	0.007	0.112	0.014
	Markers 2,3	0.004	0.001	0.012	0.003	0.022	0.004	0.058	0.009
	One-marker MA	0.012	0.003	0.034	0.005	0.060	0.008	0.123	0.016
	Two-marker MA	0.006	0.002	0.014	0.003	0.025	0.004	0.060	0.009
	All-marker JM	0.004	0.001	0.012	0.003	0.022	0.004	0.059	0.009

Table B. 4: Simulation results for Scenario 1 (D.1) with $\boldsymbol{\alpha} = (-0.5, -0.5, -0.5)$ and dependent markers. Mean and standard deviation of $\widehat{AUC}(s,t)$ and $\widehat{MSE}(s,t)$ for $s = 0, 0.5, 1, 1.5$ and $t = 0.5$ over 100 replications.

		$s=0$		$s=0.5$		$s=1$		$s=1.5$	
		Mean	SD	Mean	SD	Mean	SD	Mean	SD
$\widehat{AUC}(s,t)$	Marker 1	0.691	0.057	0.749	0.044	0.763	0.047	0.730	0.058
	Marker 2	0.691	0.058	0.742	0.038	0.758	0.056	0.726	0.067
	Marker 3	0.696	0.071	0.751	0.035	0.759	0.053	0.710	0.074
	Markers 1,2	0.719	0.058	0.783	0.041	0.811	0.050	0.786	0.059
	Markers 1,3	0.722	0.065	0.788	0.037	0.813	0.049	0.781	0.060
	Markers 2,3	0.723	0.069	0.785	0.036	0.809	0.044	0.777	0.066
	One-marker MA	0.721	0.064	0.795	0.038	0.824	0.044	0.797	0.059
	Two-marker MA	0.730	0.066	0.801	0.037	0.834	0.045	0.811	0.059
	All-marker JM	0.735	0.066	0.803	0.038	0.837	0.045	0.817	0.059
	Real value	0.738	0.066	0.808	0.038	0.839	0.045	0.820	0.059
$\widehat{MSE}(s,t)$	Marker 1	0.021	0.004	0.049	0.006	0.077	0.008	0.152	0.015
	Marker 2	0.021	0.005	0.050	0.008	0.080	0.012	0.157	0.019
	Marker 3	0.021	0.004	0.049	0.007	0.078	0.010	0.153	0.017
	Markers 1,2	0.010	0.003	0.023	0.005	0.036	0.007	0.085	0.015
	Markers 1,3	0.010	0.002	0.023	0.003	0.035	0.004	0.081	0.011
	Markers 2,3	0.011	0.004	0.024	0.006	0.037	0.009	0.085	0.016
	One-marker MA	0.018	0.004	0.041	0.006	0.064	0.009	0.140	0.014
	Two-marker MA	0.009	0.002	0.018	0.003	0.028	0.004	0.075	0.010
	All-marker JM	0.005	0.001	0.010	0.002	0.015	0.002	0.042	0.008

Table B. 5: Simulation results for Scenario 1 (D.2) with $\boldsymbol{\alpha} = (0, -0.5, -0.5)$ and dependent markers. Mean and standard deviation of $\widehat{AUC}(s,t)$ and $\widehat{MSE}(s,t)$ for $s = 0, 0.5, 1, 1.5$ and $t = 0.5$ over 100 replications.

		$s=0$		$s=0.5$		$s=1$		$s=1.5$	
		Mean	SD	Mean	SD	Mean	SD	Mean	SD
$\widehat{AUC}(s,t)$	Marker 1	0.591	0.067	0.649	0.066	0.686	0.064	0.661	0.068
	Marker 2	0.645	0.069	0.695	0.068	0.743	0.064	0.745	0.055
	Marker 3	0.647	0.060	0.698	0.060	0.746	0.056	0.743	0.050
	Markers 1,2	0.648	0.071	0.701	0.070	0.751	0.064	0.749	0.060
	Markers 1,3	0.647	0.061	0.701	0.063	0.754	0.059	0.751	0.053
	Markers 2,3	0.668	0.064	0.724	0.066	0.784	0.062	0.799	0.053
	One-marker MA	0.660	0.066	0.721	0.065	0.777	0.062	0.789	0.056
	Two-marker MA	0.665	0.061	0.723	0.066	0.784	0.063	0.799	0.053
	All-marker JM	0.667	0.064	0.724	0.067	0.784	0.062	0.800	0.054
	Real value	0.672	0.064	0.725	0.067	0.786	0.063	0.803	0.054
$\widehat{MSE}(s,t)$	Marker 1	0.008	0.002	0.030	0.004	0.061	0.009	0.128	0.018
	Marker 2	0.005	0.001	0.016	0.003	0.034	0.006	0.083	0.013
	Marker 3	0.005	0.001	0.017	0.003	0.035	0.008	0.083	0.015
	Markers 1,2	0.004	0.001	0.014	0.002	0.028	0.005	0.071	0.011
	Markers 1,3	0.004	0.001	0.014	0.002	0.029	0.006	0.072	0.012
	Markers 2,3	0.002	0.001	0.005	0.001	0.011	0.003	0.037	0.007
	One-marker MA	0.004	0.001	0.013	0.003	0.027	0.007	0.075	0.013
	Two-marker MA	0.002	0.001	0.006	0.002	0.012	0.004	0.037	0.008
	All-marker JM	0.002	0.001	0.005	0.001	0.010	0.003	0.036	0.007

Table B. 6: Simulation results for Scenario 1 (D.3) with $\boldsymbol{\alpha} = (0, -0.5, -1)$ and dependent markers. Mean and standard deviation of $\widehat{AUC}(s,t)$ and $\widehat{MSE}(s,t)$ for $s = 0, 0.5, 1, 1.5$ and $t = 0.5$ over 100 replications.

		$s=0$		$s=0.5$		$s=1$		$s=1.5$	
		Mean	SD	Mean	SD	Mean	SD	Mean	SD
$\widehat{AUC}(s,t)$	Marker 1	0.623	0.050	0.696	0.042	0.681	0.046	0.642	0.077
	Marker 2	0.672	0.059	0.736	0.040	0.724	0.053	0.711	0.070
	Marker 3	0.713	0.052	0.794	0.036	0.789	0.038	0.780	0.054
	Markers 1,2	0.678	0.054	0.749	0.038	0.738	0.049	0.719	0.069
	Markers 1,3	0.713	0.053	0.798	0.035	0.796	0.038	0.783	0.058
	Markers 2,3	0.729	0.053	0.813	0.035	0.818	0.041	0.822	0.056
	One-marker MA	0.723	0.052	0.807	0.035	0.812	0.040	0.804	0.059
	Two-marker MA	0.727	0.053	0.813	0.035	0.817	0.041	0.822	0.057
	All-marker JM	0.726	0.054	0.813	0.035	0.817	0.041	0.821	0.057
	Real value	0.728	0.054	0.821	0.034	0.821	0.043	0.823	0.059
$\widehat{MSE}(s,t)$	Marker 1	0.033	0.005	0.077	0.009	0.115	0.011	0.209	0.022
	Marker 2	0.027	0.004	0.060	0.008	0.090	0.010	0.173	0.018
	Marker 3	0.014	0.003	0.032	0.005	0.052	0.008	0.115	0.015
	Markers 1,2	0.023	0.004	0.051	0.007	0.074	0.008	0.146	0.017
	Markers 1,3	0.012	0.002	0.026	0.004	0.040	0.006	0.093	0.014
	Markers 2,3	0.007	0.001	0.015	0.002	0.023	0.003	0.060	0.010
	One-marker MA	0.015	0.003	0.033	0.005	0.053	0.008	0.121	0.015
	Two-marker MA	0.008	0.002	0.016	0.003	0.025	0.003	0.062	0.011
	All-marker JM	0.006	0.001	0.014	0.002	0.021	0.003	0.057	0.010

Table B. 7: Simulation results for Scenario 2 (M.1) with $\boldsymbol{\alpha} = (-0.5, -0.5, -0.5)$. Mean and standard deviation of $\widehat{AUC}(s,t)$ and $\widehat{MSE}(s,t)$ for $s = 0, 0.5, 1, 1.5$ and $t = 0.5$ over 100 replications.

		$s=0$		$s=0.5$		$s=1$		$s=1.5$	
		Mean	SD	Mean	SD	Mean	SD	Mean	SD
$\widehat{AUC}(s,t)$	Marker 1	0.694	0.050	0.754	0.044	0.750	0.049	0.716	0.062
	Marker 2	0.688	0.053	0.765	0.042	0.752	0.053	0.721	0.057
	Marker 3	0.596	0.049	0.661	0.044	0.684	0.059	0.677	0.068
	Markers 1,2	0.723	0.052	0.802	0.040	0.804	0.047	0.780	0.054
	Markers 1,3	0.703	0.051	0.769	0.041	0.781	0.053	0.758	0.067
	Markers 2,3	0.699	0.053	0.779	0.041	0.781	0.052	0.763	0.059
	One-marker MA	0.715	0.051	0.797	0.040	0.804	0.046	0.774	0.058
	Two-marker MA	0.724	0.053	0.805	0.040	0.812	0.047	0.794	0.056
	All-marker JM	0.728	0.053	0.809	0.039	0.819	0.048	0.803	0.057
	Real value	0.742	0.056	0.827	0.042	0.838	0.047	0.819	0.056
$\widehat{MSE}(s,t)$	Marker 1	0.021	0.004	0.048	0.007	0.078	0.009	0.158	0.016
	Marker 2	0.021	0.004	0.048	0.007	0.076	0.008	0.155	0.018
	Marker 3	0.036	0.006	0.088	0.011	0.154	0.017	0.258	0.022
	Markers 1,2	0.010	0.002	0.023	0.004	0.035	0.004	0.084	0.011
	Markers 1,3	0.018	0.003	0.040	0.006	0.068	0.010	0.144	0.016
	Markers 2,3	0.018	0.004	0.041	0.005	0.068	0.009	0.143	0.018
	One-marker MA	0.018	0.004	0.042	0.006	0.067	0.008	0.152	0.017
	Two-marker MA	0.011	0.002	0.025	0.004	0.040	0.005	0.099	0.015
	All-marker JM	0.009	0.002	0.019	0.003	0.030	0.004	0.075	0.010

Table B. 8: Simulation results for Scenario 2 (M.2) with $\boldsymbol{\alpha} = (0, -0.5, -0.5)$. Mean and standard deviation of $\widehat{AUC}(s,t)$ and $\widehat{MSE}(s,t)$ for $s = 0, 0.5, 1, 1.5$ and $t = 0.5$ over 100 replications.

		$s=0$		$s=0.5$		$s=1$		$s=1.5$	
		Mean	SD	Mean	SD	Mean	SD	Mean	SD
$\widehat{AUC}(s,t)$	Marker 1	0.587	0.064	0.657	0.052	0.676	0.056	0.680	0.066
	Marker 2	0.636	0.062	0.706	0.054	0.753	0.053	0.743	0.061
	Marker 3	0.589	0.056	0.647	0.047	0.689	0.054	0.701	0.047
	Markers 1,2	0.637	0.067	0.710	0.052	0.755	0.052	0.752	0.062
	Markers 1,3	0.612	0.061	0.684	0.052	0.716	0.053	0.726	0.053
	Markers 2,3	0.647	0.058	0.719	0.052	0.773	0.056	0.776	0.056
	One-marker MA	0.635	0.063	0.709	0.054	0.760	0.052	0.754	0.061
	Two-marker MA	0.642	0.066	0.718	0.051	0.767	0.052	0.771	0.058
	All-marker JM	0.646	0.064	0.721	0.050	0.773	0.056	0.776	0.057
	Real value	0.648	0.070	0.742	0.047	0.792	0.056	0.796	0.052
$\widehat{MSE}(s,t)$	Marker 1	0.008	0.002	0.029	0.005	0.060	0.006	0.124	0.013
	Marker 2	0.005	0.001	0.016	0.003	0.034	0.005	0.081	0.011
	Marker 3	0.009	0.003	0.033	0.006	0.075	0.011	0.150	0.016
	Markers 1,2	0.004	0.001	0.014	0.002	0.028	0.004	0.070	0.010
	Markers 1,3	0.007	0.002	0.024	0.004	0.050	0.006	0.113	0.012
	Markers 2,3	0.004	0.001	0.012	0.002	0.026	0.004	0.070	0.010
	One-marker MA	0.005	0.001	0.017	0.003	0.036	0.005	0.089	0.014
	Two-marker MA	0.004	0.001	0.013	0.002	0.028	0.004	0.070	0.010
	All-marker JM	0.003	0.001	0.011	0.002	0.023	0.004	0.063	0.010

Table B. 9: Simulation results for Scenario 2 (M.3) with $\boldsymbol{\alpha} = (0, -0.5, -1)$. Mean and standard deviation of $\widehat{AUC}(s,t)$ and $\widehat{MSE}(s,t)$ for $s = 0, 0.5, 1, 1.5$ and $t = 0.5$ over 100 replications.

		$s = 0$		$s = 0.5$		$s = 1$		$s = 1.5$	
		Mean	SD	Mean	SD	Mean	SD	Mean	SD
$\widehat{AUC}(s,t)$	Marker 1	0.635	0.055	0.683	0.050	0.674	0.061	0.651	0.079
	Marker 2	0.680	0.048	0.727	0.050	0.733	0.069	0.703	0.074
	Marker 3	0.616	0.046	0.680	0.045	0.694	0.051	0.716	0.062
	Markers 1,2	0.689	0.049	0.741	0.051	0.748	0.066	0.719	0.070
	Markers 1,3	0.665	0.051	0.726	0.048	0.733	0.051	0.735	0.061
	Markers 2,3	0.698	0.045	0.760	0.046	0.777	0.054	0.782	0.063
	One-marker MA	0.683	0.048	0.740	0.050	0.758	0.064	0.744	0.072
	Two-marker MA	0.695	0.050	0.758	0.047	0.775	0.058	0.768	0.067
	All-marker JM	0.701	0.050	0.764	0.049	0.781	0.054	0.783	0.063
	Real value	0.744	0.050	0.807	0.048	0.828	0.049	0.828	0.054
$\widehat{MSE}(s,t)$	Marker 1	0.035	0.006	0.079	0.008	0.114	0.011	0.201	0.020
	Marker 2	0.028	0.005	0.061	0.008	0.088	0.010	0.165	0.017
	Marker 3	0.040	0.007	0.088	0.010	0.148	0.017	0.244	0.022
	Markers 1,2	0.024	0.004	0.052	0.007	0.073	0.008	0.140	0.017
	Markers 1,3	0.029	0.006	0.064	0.008	0.102	0.014	0.189	0.021
	Markers 2,3	0.022	0.005	0.047	0.007	0.077	0.014	0.152	0.021
	One-marker MA	0.028	0.005	0.063	0.008	0.096	0.012	0.186	0.021
	Two-marker MA	0.023	0.004	0.049	0.007	0.076	0.011	0.151	0.019
	All-marker JM	0.019	0.004	0.041	0.006	0.064	0.012	0.132	0.021

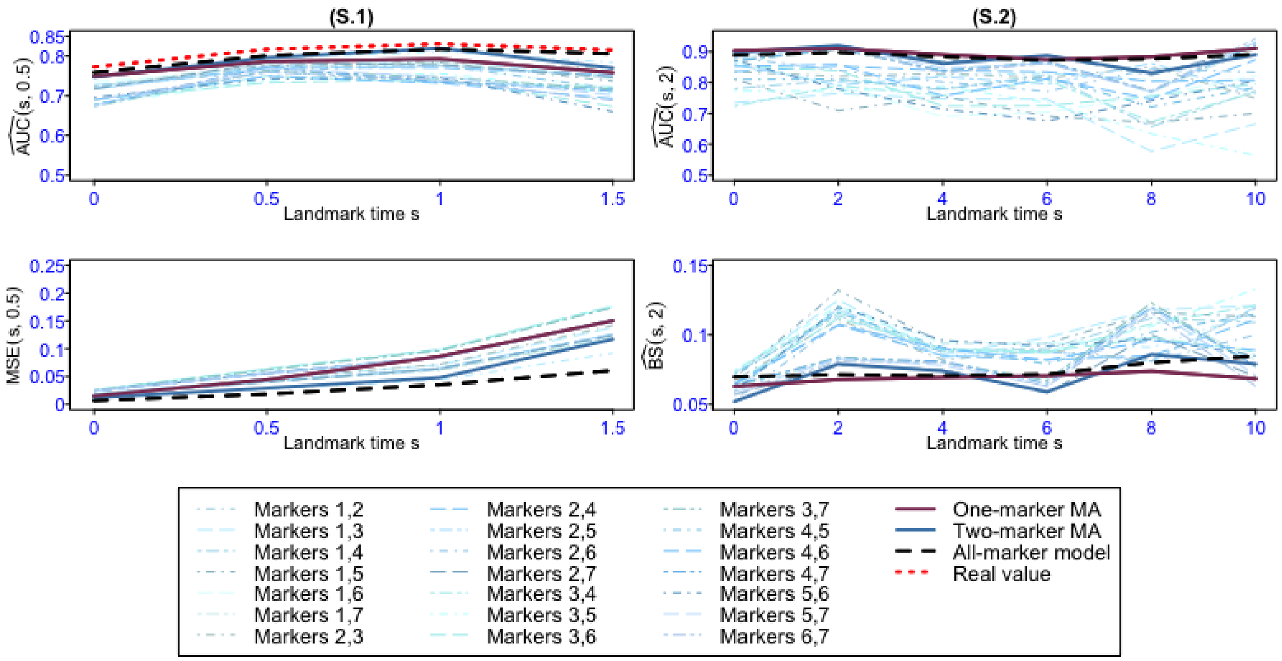

Figure B. 1: $\widehat{AUC}(s,t)$ (first row), $\widehat{BS}(s,t)$ or $\widehat{MSE}(s,t)$ (second row) for Scenario 3 over 100 replications. S.1: $\boldsymbol{\alpha} = (-0.5, -0.5, -0.5, 0, 0, 0, 0)$ and S.2: $\boldsymbol{\alpha} = (-0.5, -0.5, -1, 0, 0, 0, 0)$ and dependent markers with landmark times $s = 0, 0.5, 1, 1.5$ and prediction windows of $t = 0.5$. S.3: mimicking the PBC2 data by using bootstrap simulation with landmark times $s = 0, 2, 4, 6, 8, 10$ and prediction windows of $t = 2$ over 100 replications.

Table B. 10: Simulation results for Scenario 3 (S.1) with $\boldsymbol{\alpha} = (-0.5, -0.5, -0.5, 0, 0, 0, 0)$ and dependent markers. Mean and standard deviation of $\widehat{AUC}(s,t)$ for $s = 0, 0.5, 1, 1.5$ and $t = 0.5$ over 100 replications.

	$s = 0$		$s = 0.5$		$s = 1$		$s = 1.5$	
	Mean	SD	Mean	SD	Mean	SD	Mean	SD
Marker 1	0.718	0.046	0.757	0.054	0.767	0.080	0.719	0.135
Marker 2	0.712	0.062	0.749	0.059	0.751	0.055	0.723	0.102
Marker 3	0.629	0.057	0.671	0.071	0.685	0.086	0.664	0.174
Marker 4	0.657	0.074	0.735	0.063	0.695	0.071	0.707	0.118
Marker 5	0.655	0.061	0.717	0.069	0.715	0.078	0.671	0.145
Marker 6	0.676	0.055	0.706	0.054	0.701	0.087	0.617	0.164
Marker 7	0.651	0.042	0.747	0.068	0.719	0.087	0.681	0.139
Markers 1,2	0.749	0.056	0.794	0.061	0.811	0.076	0.784	0.115
Markers 1,3	0.727	0.049	0.773	0.060	0.791	0.080	0.746	0.145
Markers 1,4	0.724	0.062	0.777	0.064	0.777	0.075	0.750	0.119
Markers 1,5	0.721	0.052	0.775	0.066	0.784	0.073	0.737	0.128
Markers 1,6	0.735	0.050	0.768	0.057	0.780	0.081	0.715	0.166
Markers 1,7	0.721	0.048	0.786	0.053	0.784	0.067	0.743	0.143
Markers 2,3	0.721	0.066	0.766	0.065	0.787	0.070	0.768	0.108
Markers 2,4	0.718	0.065	0.771	0.060	0.770	0.066	0.752	0.097
Markers 2,5	0.717	0.064	0.768	0.063	0.776	0.061	0.749	0.101
Markers 2,6	0.724	0.065	0.763	0.060	0.776	0.062	0.718	0.105
Markers 2,7	0.717	0.059	0.783	0.059	0.778	0.054	0.747	0.102
Markers 3,4	0.678	0.071	0.750	0.064	0.733	0.073	0.722	0.130
Markers 3,5	0.678	0.064	0.739	0.076	0.756	0.078	0.707	0.148
Markers 3,6	0.690	0.061	0.733	0.071	0.742	0.084	0.673	0.157
Markers 3,7	0.673	0.047	0.763	0.070	0.747	0.070	0.716	0.135
Markers 4,5	0.677	0.068	0.761	0.062	0.736	0.085	0.718	0.112
Markers 4,6	0.690	0.062	0.749	0.058	0.732	0.075	0.693	0.134
Markers 4,7	0.676	0.063	0.772	0.064	0.735	0.070	0.712	0.125
Markers 5,6	0.696	0.055	0.743	0.062	0.746	0.070	0.660	0.143
Markers 5,7	0.675	0.048	0.769	0.071	0.748	0.069	0.702	0.144
Markers 6,7	0.690	0.049	0.759	0.062	0.740	0.071	0.688	0.145
One-marker MA	0.741	0.057	0.789	0.060	0.811	0.069	0.745	0.115
Two-marker MA	0.747	0.058	0.796	0.058	0.820	0.071	0.770	0.122
All-marker JM	0.751	0.059	0.807	0.064	0.828	0.072	0.797	0.116
Real value	0.763	0.062	0.828	0.059	0.838	0.077	0.807	0.129

Table B. 11: Simulation results for Scenario 3 (S.1) with $\boldsymbol{\alpha} = (-0.5, -0.5, -0.5, 0, 0, 0, 0)$ and dependent markers. Mean and standard deviation of $\widehat{MSE}(s,t)$ for $s = 0, 0.5, 1, 1.5$ and $t = 0.5$ over 100 replications.

	$s = 0$		$s = 0.5$		$s = 1$		$s = 1.5$	
	Mean	SD	Mean	SD	Mean	SD	Mean	SD
Marker 1	0.021	0.003	0.051	0.006	0.079	0.008	0.146	0.025
Marker 2	0.020	0.004	0.051	0.006	0.083	0.008	0.154	0.019
Marker 3	0.034	0.006	0.089	0.010	0.143	0.014	0.237	0.027
Marker 4	0.028	0.004	0.070	0.008	0.107	0.013	0.184	0.029
Marker 5	0.028	0.005	0.070	0.009	0.109	0.011	0.184	0.024
Marker 6	0.028	0.005	0.071	0.009	0.110	0.010	0.185	0.022
Marker 7	0.028	0.005	0.069	0.007	0.108	0.012	0.182	0.024
Markers 1,2	0.011	0.002	0.027	0.004	0.044	0.005	0.092	0.019
Markers 1,3	0.019	0.003	0.046	0.006	0.071	0.007	0.138	0.022
Markers 1,4	0.018	0.002	0.041	0.005	0.062	0.007	0.119	0.024
Markers 1,5	0.018	0.003	0.041	0.005	0.063	0.006	0.121	0.022
Markers 1,6	0.017	0.003	0.041	0.005	0.063	0.007	0.120	0.020
Markers 1,7	0.017	0.003	0.040	0.005	0.062	0.008	0.118	0.023
Markers 2,3	0.017	0.004	0.045	0.006	0.072	0.007	0.142	0.019
Markers 2,4	0.017	0.004	0.041	0.005	0.064	0.008	0.126	0.018
Markers 2,5	0.017	0.004	0.041	0.006	0.065	0.007	0.126	0.019
Markers 2,6	0.017	0.004	0.042	0.005	0.065	0.006	0.125	0.017
Markers 2,7	0.016	0.003	0.040	0.005	0.064	0.007	0.124	0.018
Markers 3,4	0.026	0.004	0.063	0.007	0.097	0.011	0.175	0.026
Markers 3,5	0.026	0.005	0.063	0.009	0.098	0.011	0.176	0.024
Markers 3,6	0.025	0.005	0.064	0.008	0.099	0.009	0.177	0.022
Markers 3,7	0.025	0.004	0.062	0.007	0.097	0.010	0.174	0.023
Markers 4,5	0.024	0.004	0.056	0.007	0.084	0.010	0.151	0.024
Markers 4,6	0.024	0.004	0.057	0.007	0.085	0.011	0.152	0.025
Markers 4,7	0.024	0.004	0.055	0.006	0.084	0.011	0.150	0.025
Markers 5,6	0.024	0.005	0.056	0.008	0.085	0.009	0.150	0.021
Markers 5,7	0.024	0.004	0.055	0.006	0.085	0.010	0.150	0.022
Markers 6,7	0.024	0.005	0.056	0.007	0.085	0.009	0.149	0.021
One-marker MA	0.018	0.003	0.046	0.006	0.074	0.008	0.158	0.027
Two-marker MA	0.012	0.002	0.029	0.005	0.048	0.006	0.117	0.031
All-marker JM	0.008	0.002	0.019	0.003	0.030	0.004	0.064	0.016

Table B. 12: Simulation results for Scenario 3 (S.2) mimicking PBC2 data. Mean and standard deviation of $\widehat{AUC}(s,t)$ for $s = 0, 2, 4, 6, 8, 10$ and $t = 2$ over 100 bootstrap samples.

	$s=0$		$s=2$		$s=4$		$s=6$		$s=8$		$s=10$	
	Mean	SD	Mean	SD	Mean	SD	Mean	SD	Mean	SD	Mean	SD
Marker 1	0.801	0.046	0.747	0.016	0.719	0.049	0.676	0.054	0.659	0.065	0.682	0.094
Marker 2	0.834	0.027	0.805	0.033	0.827	0.035	0.768	0.057	0.890	0.051	0.852	0.111
Marker 3	0.696	0.063	0.718	0.040	0.618	0.090	0.661	0.075	0.559	0.088	0.450	0.174
Marker 4	0.757	0.065	0.826	0.031	0.719	0.065	0.810	0.042	0.709	0.048	0.760	0.134
Marker 5	0.753	0.072	0.657	0.029	0.686	0.026	0.599	0.040	0.727	0.066	0.722	0.126
Marker 6	0.872	0.035	0.802	0.027	0.676	0.044	0.686	0.047	0.740	0.071	0.863	0.158
Marker 7	0.861	0.054	0.931	0.021	0.857	0.043	0.867	0.063	0.811	0.035	0.910	0.032
Markers 1,2	0.856	0.053	0.801	0.024	0.823	0.036	0.830	0.061	0.807	0.070	0.805	0.052
Markers 1,3	0.734	0.073	0.765	0.028	0.747	0.076	0.746	0.033	0.577	0.125	0.666	0.096
Markers 1,4	0.780	0.046	0.808	0.045	0.777	0.071	0.826	0.039	0.657	0.078	0.773	0.088
Markers 1,5	0.811	0.073	0.709	0.050	0.755	0.062	0.691	0.082	0.670	0.054	0.700	0.089
Markers 1,6	0.868	0.029	0.788	0.036	0.690	0.077	0.704	0.066	0.756	0.095	0.822	0.102
Markers 1,7	0.877	0.046	0.909	0.027	0.840	0.054	0.869	0.013	0.768	0.074	0.913	0.040
Markers 2,3	0.835	0.060	0.820	0.008	0.829	0.023	0.815	0.075	0.850	0.060	0.749	0.096
Markers 2,4	0.835	0.071	0.853	0.023	0.839	0.016	0.834	0.078	0.804	0.095	0.808	0.053
Markers 2,5	0.859	0.057	0.791	0.021	0.819	0.030	0.795	0.088	0.862	0.055	0.765	0.078
Markers 2,6	0.882	0.054	0.839	0.026	0.802	0.034	0.771	0.082	0.845	0.065	0.833	0.066
Markers 2,7	0.886	0.063	0.921	0.026	0.868	0.038	0.886	0.042	0.824	0.076	0.916	0.015
Markers 3,4	0.723	0.041	0.789	0.029	0.758	0.072	0.808	0.068	0.673	0.071	0.769	0.060
Markers 3,5	0.773	0.082	0.772	0.019	0.787	0.057	0.735	0.085	0.634	0.072	0.564	0.110
Markers 3,6	0.855	0.024	0.838	0.017	0.724	0.081	0.725	0.082	0.756	0.080	0.795	0.088
Markers 3,7	0.869	0.051	0.908	0.028	0.837	0.050	0.864	0.025	0.773	0.078	0.930	0.039
Markers 4,5	0.810	0.046	0.811	0.044	0.780	0.044	0.817	0.085	0.721	0.086	0.784	0.063
Markers 4,6	0.852	0.040	0.859	0.039	0.757	0.058	0.812	0.069	0.747	0.091	0.874	0.042
Markers 4,7	0.869	0.045	0.911	0.029	0.837	0.050	0.868	0.029	0.772	0.077	0.923	0.040
Markers 5,6	0.873	0.036	0.779	0.039	0.714	0.051	0.676	0.091	0.741	0.095	0.806	0.105
Markers 5,7	0.881	0.038	0.918	0.031	0.846	0.044	0.862	0.026	0.769	0.074	0.931	0.037
Markers 6,7	0.893	0.045	0.916	0.030	0.831	0.051	0.862	0.028	0.772	0.091	0.942	0.044
One-marker MA	0.886	0.041	0.949	0.021	0.879	0.038	0.861	0.061	0.878	0.034	0.920	0.059
Two-marker MA	0.898	0.054	0.920	0.029	0.861	0.039	0.888	0.032	0.831	0.083	0.890	0.080
All-marker JM	0.873	0.048	0.932	0.019	0.870	0.040	0.863	0.065	0.877	0.067	0.895	0.047

Table B. 13: Simulation results for Scenario 3 (S.2) mimicking PBC2 data. Mean and standard deviation of $\widehat{BS}(s,t)$ for $s = 0, 2, 4, 6, 8, 10$ and $t = 2$ over 100 bootstrap samples.

	$s=0$		$s=2$		$s=4$		$s=6$		$s=8$		$s=10$	
	Mean	SD	Mean	SD	Mean	SD	Mean	SD	Mean	SD	Mean	SD
Marker 1	0.086	0.029	0.130	0.026	0.095	0.017	0.092	0.019	0.138	0.037	0.117	0.038
Marker 2	0.076	0.024	0.121	0.023	0.086	0.017	0.091	0.017	0.090	0.030	0.090	0.030
Marker 3	0.091	0.031	0.124	0.024	0.094	0.017	0.090	0.018	0.131	0.033	0.132	0.044
Marker 4	0.088	0.031	0.107	0.020	0.094	0.017	0.075	0.014	0.126	0.032	0.111	0.043
Marker 5	0.090	0.032	0.134	0.030	0.103	0.018	0.091	0.016	0.119	0.027	0.110	0.036
Marker 6	0.074	0.024	0.117	0.019	0.096	0.018	0.084	0.017	0.127	0.040	0.079	0.029
Marker 7	0.072	0.022	0.075	0.018	0.075	0.015	0.055	0.013	0.126	0.031	0.091	0.033
Markers 1,2	0.062	0.031	0.118	0.013	0.084	0.021	0.094	0.023	0.091	0.033	0.116	0.038
Markers 1,3	0.072	0.036	0.125	0.013	0.086	0.022	0.098	0.019	0.118	0.027	0.121	0.036
Markers 1,4	0.072	0.036	0.114	0.015	0.088	0.023	0.090	0.023	0.112	0.029	0.109	0.029
Markers 1,5	0.069	0.034	0.132	0.018	0.094	0.023	0.094	0.018	0.116	0.026	0.113	0.031
Markers 1,6	0.061	0.029	0.120	0.015	0.094	0.023	0.094	0.021	0.098	0.034	0.085	0.020
Markers 1,7	0.062	0.030	0.082	0.018	0.080	0.018	0.068	0.012	0.119	0.029	0.082	0.019
Markers 2,3	0.067	0.035	0.111	0.009	0.082	0.021	0.089	0.023	0.082	0.026	0.119	0.050
Markers 2,4	0.064	0.033	0.107	0.017	0.085	0.021	0.082	0.024	0.085	0.032	0.111	0.040
Markers 2,5	0.059	0.030	0.119	0.014	0.084	0.021	0.088	0.020	0.081	0.028	0.120	0.049
Markers 2,6	0.053	0.027	0.108	0.014	0.088	0.022	0.088	0.019	0.081	0.032	0.100	0.032
Markers 2,7	0.057	0.029	0.078	0.016	0.075	0.016	0.058	0.010	0.100	0.039	0.070	0.020
Markers 3,4	0.074	0.037	0.116	0.012	0.089	0.025	0.087	0.026	0.108	0.025	0.121	0.034
Markers 3,5	0.074	0.037	0.121	0.013	0.089	0.023	0.088	0.018	0.106	0.022	0.133	0.058
Markers 3,6	0.065	0.031	0.112	0.012	0.090	0.022	0.088	0.019	0.098	0.030	0.088	0.027
Markers 3,7	0.063	0.029	0.084	0.018	0.080	0.018	0.067	0.011	0.123	0.034	0.077	0.023
Markers 4,5	0.071	0.034	0.116	0.016	0.091	0.023	0.079	0.022	0.102	0.027	0.115	0.036
Markers 4,6	0.065	0.031	0.107	0.018	0.090	0.022	0.082	0.019	0.096	0.033	0.089	0.022
Markers 4,7	0.064	0.030	0.083	0.019	0.081	0.018	0.065	0.012	0.120	0.031	0.078	0.023
Markers 5,6	0.059	0.027	0.120	0.015	0.096	0.023	0.092	0.019	0.097	0.031	0.085	0.020
Markers 5,7	0.059	0.028	0.083	0.019	0.078	0.017	0.063	0.011	0.119	0.034	0.071	0.019
Markers 6,7	0.057	0.027	0.081	0.020	0.080	0.017	0.063	0.010	0.116	0.037	0.063	0.019
One-marker MA	0.061	0.019	0.071	0.018	0.072	0.015	0.057	0.014	0.090	0.022	0.062	0.023
Two-marker MA	0.052	0.026	0.079	0.017	0.074	0.014	0.059	0.009	0.086	0.034	0.079	0.012
All-marker JM	0.068	0.022	0.075	0.017	0.074	0.015	0.057	0.015	0.093	0.032	0.081	0.022

Table B. 14: Simulation results for Scenario 4. Mean and standard deviation of $\widehat{AUC}(s,t)$ and $\widehat{BS}(s,t)$ for $s = 0, 0.5, 1, 1.5$ and $t = 0.5$ over 100 replications.

		$s=0$		$s=0.5$		$s=1$		$s=1.5$	
		Mean	SD	Mean	SD	Mean	SD	Mean	SD
$\widehat{AUC}(s,t)$	Marker 1	0.645	0.035	0.621	0.040	0.603	0.052	0.505	0.043
	Marker 2	0.531	0.036	0.584	0.042	0.647	0.038	0.728	0.047
	One-marker MA	0.644	0.034	0.634	0.038	0.660	0.045	0.728	0.047
	All-marker JM	0.611	0.036	0.638	0.037	0.675	0.042	0.679	0.041
$\widehat{BS}(s,t)$	Marker 1	0.104	0.011	0.148	0.015	0.200	0.017	0.270	0.029
	Marker 2	0.107	0.012	0.150	0.015	0.194	0.016	0.232	0.026
	One-marker MA	0.104	0.011	0.148	0.015	0.194	0.016	0.232	0.026
	All-marker JM	0.105	0.012	0.147	0.015	0.190	0.016	0.239	0.028

Table B. 15: The coverage rates (CR) and the length (Length) of the 95% credible interval of the predicted probabilities obtained from the all-marker joint model estimated by `JMbayes` and of the 95% confidence intervals based on Buckland et al. (1997)'s approach over 100 replications. M.1: two Gaussian and one binary markers, $\boldsymbol{\alpha} = (-0.5, -0.5, -0.5)$ and dependent markers, M.2: two Gaussian and one binary markers, $\boldsymbol{\alpha} = (0, -0.5, -0.5)$ and dependent markers and M.3: two Gaussian and one binary markers, $\boldsymbol{\alpha} = (0, -0.5, -1)$ and dependent markers.

		$s = 0$		$s = 0.5$		$s = 1$		$s = 1.5$	
		CR	Length	CR	Length	CR	Length	CR	Length
M.1	One-marker MA	0.948	0.373	0.944	0.594	0.917	0.689	0.786	0.598
	Two-marker MA	0.952	0.399	0.977	0.638	0.957	0.740	0.882	0.663
	All-marker JM	0.690	0.155	0.709	0.253	0.753	0.317	0.793	0.346
M.2	One-marker MA	0.948	0.222	0.924	0.405	0.930	0.555	0.852	0.507
	Two-marker MA	0.971	0.246	0.983	0.435	0.963	0.582	0.922	0.561
	All-marker JM	0.757	0.107	0.699	0.178	0.726	0.254	0.814	0.273
M.3	One-marker MA	0.889	0.366	0.916	0.563	0.916	0.642	0.788	0.536
	Two-marker MA	0.967	0.414	0.971	0.620	0.948	0.698	0.904	0.635
	All-marker JM	0.729	0.239	0.747	0.332	0.802	0.396	0.794	0.392
